# Do orthologous gene phylogenies really support tree-thinking?

**DOI:** 10.1186/1471-2148-5-33

**Published:** 2005-05-24

**Authors:** E Bapteste, E Susko, J Leigh, D MacLeod, RL Charlebois, WF Doolittle

**Affiliations:** 1 GenomeAtlantic, 1721 Lower Water Street, Suite 401, Halifax, NS, B3J 1S5, Canada; 2 Dalhousie University, Department of Biochemistry & Molecular Biology, 5850 College St., Halifax, NS, B3H 1X5, Canada; 3 Dalhousie University, Department of Mathematics and Statistics, Halifax, Nova Scotia, Canada

## Abstract

**Background:**

Since Darwin's Origin of Species, reconstructing the Tree of Life has been a goal of evolutionists, and tree-thinking has become a major concept of evolutionary biology. Practically, building the Tree of Life has proven to be tedious. Too few morphological characters are useful for conducting conclusive phylogenetic analyses at the highest taxonomic level. Consequently, molecular sequences (genes, proteins, and genomes) likely constitute the only useful characters for constructing a phylogeny of all life. For this reason, tree-makers expect a lot from gene comparisons. The simultaneous study of the largest number of molecular markers possible is sometimes considered to be one of the best solutions in reconstructing the genealogy of organisms. This conclusion is a direct consequence of tree-thinking: if gene inheritance conforms to a tree-like model of evolution, sampling more of these molecules will provide enough phylogenetic signal to build the Tree of Life. The selection of congruent markers is thus a fundamental step in simultaneous analysis of many genes.

**Results:**

Heat map analyses were used to investigate the congruence of orthologues in four datasets (archaeal, bacterial, eukaryotic and alpha-proteobacterial). We conclude that we simply cannot determine if a large portion of the genes have a common history. In addition, none of these datasets can be considered free of lateral gene transfer.

**Conclusion:**

Our phylogenetic analyses do not support tree-thinking. These results have important conceptual and practical implications. We argue that representations other than a tree should be investigated in this case because a non-critical concatenation of markers could be highly misleading.

## Background

Tree-thinking, the explanation of evolutionary events in the context of a tree, has inspired many philosophers and evolutionists [1]. Some tree-thinkers classically employed this pattern, labelling it the "organismal tree," and arguing that it depicts the dividing pattern of cells, the path of the envelope division of living beings through time [2] (if no cell-cell fusion occurs [3]). Other authors, even if they have retained this drawing to describe evolution, have redefined the meaning of the tree as a "prevailing trend in the evolution of genome-scale gene sets rather than as a complete picture of evolution" [4]. In any case, the reconstruction of the vertical history is decisive and relies on defining sets of congruent characters [5-7]. At the morphological level, such comparable characters are hardly identifiable. In prokaryotes, it is only with the advance of molecular phylogenetics that classification has experienced a hopeful yet limited rebound [8]. For a tree-thinker, the use of orthologue genes could unite practical and conceptual advantages. They allow us to describe the organism from the molecules, because they fit perfectly within the traditional approach of molecular phylogenetics for which the history of genes tells the history of species. They provide a vast quantity of comparable characters, and since they have been inherited from ancestor to descendants, they should likely be congruent, retracing the history of species diversification [9]. Such ideal markers are needed to reconstruct a convincing phylogenetic tree, if the tree is the right model for representing evolution.

In practice, the identification of congruent genes is mostly based on exclusion of potentially incongruent markers (i.e., paralogues, xenologues). Only broadly distributed orthologues are generally retained, if their individual phylogenies do not support apparently odd relationships [7,9-11]. The set of candidate congruent markers is sometimes further tested. Genes are concatenated to maximise the phylogenetic signal they contain, and a best tree is inferred from this large dataset. Statistical approaches are then used to test whether individual markers reject this best tree. If they fail to reject it, genes are claimed to be congruent with it. If some genes reject it, they are secondarily excluded from the core. This process is repeated until the dataset stabilizes.

Generally, these successive conditions allow the retention of a small minority of the genes present in an initial set of genomes [7]. The quantity of molecular information in these genes might thus be critical in resolving ancient phylogenetic relationships. In this context, simultaneous analyses are seen as the logical solution to produce a robust tree:supertrees [12-14] or *a posteriori *consensus approaches [15] can be employed. Supertree methods assemble an input set of separate phylogenetic trees with shared taxa into a larger tree [13,16] (or several trees). By fitting variously supported clades together, they allow large phylogenies based on different characters to be constructed rapidly and have been applied to a broad range of species [17]. Consensus approaches, such as sequences concatenation [5,18] or by averaging over a large number of genes [19,20] produce resolved phylogenies by overwhelming noise with signal that is presumed to be systematically congruent and historically true, though weak.

These approaches, aiming to produce a tree-like pattern whether the tree is the right model for representing evolution or not, are derived from a tree-thinking perspective. This could, however, be flawed, and deserves criticism on conceptual and statistical grounds. First, some genes have been shown not to follow a simple model of inheritance. For instance, lateral transfers of genetic material are common in nature. All living systems from viruses [21] to eukaryotes [22] can participate in the transfer of genetic material. They occur within domains of life, but also across domains, for different markers. There is now broad general agreement that lateral gene transfer (LGT) is a major force in the evolution of prokaryotes [23-27]. Additional evidence suggests that gene transfer might also be an important evolutionary mechanism in protist evolution. Andersson et *al. *[28] recently reported that alanyl-tRNA synthetase had been transferred from Nanoarchaeota to Diplomonads and Parabasalids. The same authors [29] showed that LGT has affected both eukaryotes and prokaryotes with respect to glutamate dehydrogenase. Recombination is also an issue for tree-reconstruction. Software such as Splitstree [30] or T-Rex [31] were developed to acknowledge this. A tree-thinker may choose to ignore conflicting signal as if it was noise, even if legitimate evolutionary events underlie it [18]. However, if this "noise" is in fact *bona fide *phylogenetic signal, then maybe tree-thinking is inappropriate.

The failure of individual markers to reject a concatenation-tree [7] is not a real test that genes are congruent. There are many reasons why a single gene can fail to reject a tree issued from a concatenation, many of which do not imply that these genes are effectively in favour of this single history [32]. Briefly, the best tree of a concatenation, being an average of the weak signals and noise in many genes can be a central tendency with very low discriminative power. The fact that every gene "agrees" with such a tree does not mean the concatenation tree is true, just that it reflects a part of the signal/noise of every gene. Importantly, this apparent "agreement" is also expected if individual markers contain very little phylogenetic signal. A weak marker would fail to reject most of the test trees, not only the concatenation tree.

The relative weakness of individual markers can be tested statistically when considering not only the concatenation tree but also many different trees. If several different topologies cannot be rejected by a given gene, then, unfortunately, its phylogeny does not tell us much about its actual history. For this reason, some analyses of congruence use multiple alternative test topologies [11,33]. Such analyses describe each gene by a list of likelihood or p-values associated with a set of given topologies. These lists are summarized in a large matrix of genes and topologies, which is subsequently treated by clustering methods. For instance, in principal component analyses (PCA), each gene is represented as a point in a two-dimensional projection of its position in n-dimensional space, the coordinates for each gene in that space being related to its degree of support for each of the n tree topologies tested. Thus, genes supporting and rejecting the same sets of trees should group together, constituting a cloud, while genes with atypical support/rejection patterns of topologies should be displayed elsewhere on the PCA [10]. Most of the time, PCAs produce a central cloud containing most of the markers. From this, authors generally conclude that the markers in a cloud are congruent [10,11]. However, again, a cloud of genes in a PCA may have various explanations, which differ from each gene in a cloud supporting the same history. Genes can still be lacking signal. The set of topologies might be too restricted or biased to allow discrimination between genes: if the vast majority of the topologies are very unlikely, none of them will ever be favoured, and differential clustering is unexpected. Yet, in no case would common rejection of unlikely topologies assess that the markers are congruent. Finally, even genes with different robust signal (i.e., due to recent LGT) might cluster together on a PCA, if the set of test-topologies does not allow us to identify this relationship.

Interestingly, these features can be investigated more explicitly by an alternative statistical approach: the heat map [34]. We have thus decided to re-explore the congruence in some datasets of orthologues with this method. Briefly, heat maps (HMs) generate graphs through hierarchical or partitional clustering. They allow the simultaneous display and clustering of all combinations of genes and test conditions together [35-37]. Thus genes that have the most similar responses to topologies, and topologies that are the most similar in terms of the responses they evoke from genes, can be independently identified. More precisely, when applied to phylogenetics, "responses" are p-values for each set of genes, given those topologies. Clustering of genes allows identification of one or more set of genes that might share a common evolutionary history. Clustering of topologies allows us to identify which trees are equally or nearly equally supported, and thus to assess how many distinct "best trees" there might be for a dataset of genes.

In this paper, we investigate the phylogenetic signal of four datasets in order to address a simple question: do the phylogenies of orthologs really favour tree-thinking and thus justify attempts of tree-reconstruction? Can we be reasonably confident that their history is free of LGT? We observe that no unique common history can be established for these genes. In all cases, genes fail to favour a single tree. We also observe that some of these genes support incongruent histories. Consequently, the tree-thinking on which gene concatenations rest does not proceed from phylogenetic conclusions, nor is it *a priori *a safe phylogenetic practice. We argue that using only the robustly resolved parts of individual phylogenies without necessarily expecting a tree as a result is likely more appropriate.

## Results and discussion

### Testing the phylogenetic information of datasets of orthologues

We used heat map analyses to investigate both the congruence and the absence of LGT in four selections of orthologues, two features that would be in favour of the reconstruction of an organismal tree, recently challenged by multiple analyses of comparative genomics. Indeed, it was showed that gene gains and gene losses contributed to the evolution of a substantial majority of orthologous sets of prokaryotic proteins [38-40]. Such results suggested that the simple notion of a single Tree of Life that would accurately and definitively depict the evolution of all life forms was gone forever [4]. Wolf et *al. *[4] concluded that the concept of a tree could only be rescued by weakening its meaning, and considering it only as "a central trend in the rich patchwork of evolutionary history, replete with gene loss and horizontal transfer events".

**Figure 1:**
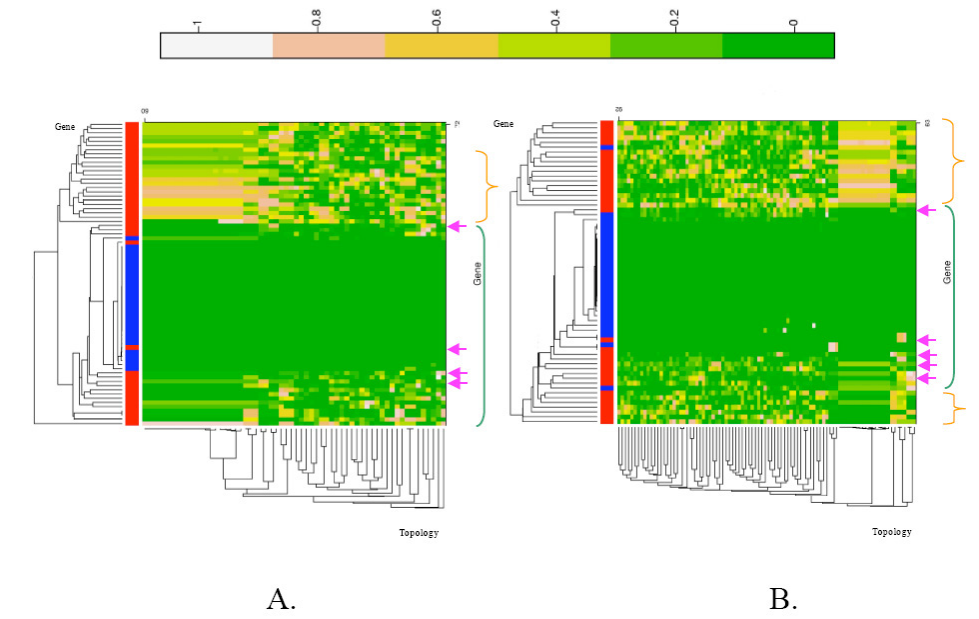
Figure 1A. displays the heat map for the archaeal dataset, Figure 1B. for the eukaryotic dataset. Heat maps include two kinds of markers: actual ones, indicated by a red rectangle at the left of the heat map, and artificial markers with extreme LGT (see main text), indicated in blue. They are based on a set of plausible topologies (see main text). The number of genes and topologies in the analysis are indicated on the heat map. These heat maps are double-clustered by genes and by topologies. The hierarchical clusters are represented by a tree of genes and a tree of topologies along the heat map. In the left band, the relative distribution of red and blue rectangles reflects the presence/absence of clustering of actual markers with artificial ones. Inside a heat map each dot of colour corresponds to the p-value for a given gene and a given topology. The p-values range from 0 (rejection) to 1 (support). The colour code associated with these p-values (from green for rejection to white for support) are reported above the heatmap. On the right of each heat map, the orange brackets indicate regions containing markers with a weak discriminatory power; the green brackets indicate regions containing markers with a stronger discriminatory power. Amongst the markers with a stronger phylogenetic signal, pink arrows point to some instances of conflicting signal in actual markers. They indicate different columns displaying a contrasting pattern of colour and contradictory p-values for several orthologues in a dataset.

To test whether the reconstruction of any organismal tree was then phylogenetically justified when simultaneously using multiple orthologues, our heat maps contain two kinds of markers: artificial ones, with up to three simulated LGT events, and actual ones. A red rectangle at the left of the heat map identifies the actual markers, while a blue rectangle indicates the artificial markers (Figure 1). For each dataset, a set of plausible topologies was selected from the study of the phylogenetic signal of the markers. These plausible topologies correspond to the trees supported by a large majority of the markers. The support was estimated as the p-values from the AU test. This support is displayed in the heat map through a colour code. Lighter colours indicate a higher probability of the data given the tree (that is, stronger support) while greener colours indicate lower probabilities (stronger rejection). Heat maps were also double-clustered to group genes with similar pattern of support/rejection along columns, and to group topologies receiving similar support/rejection along rows. These hierarchical clusters are represented by a tree of genes and a tree of topologies along the heat map. Hence, to know which and how many topologies a given gene supports, one simply needs to look along its corresponding column in the heat map. If a gene is very discriminatory and favours only a few topologies, the column will be mostly green. In contrast, a gene with a weak phylogenetic signal is unable to decide between multiple topologies and its column is mostly light-coloured.

**Figure 2:**
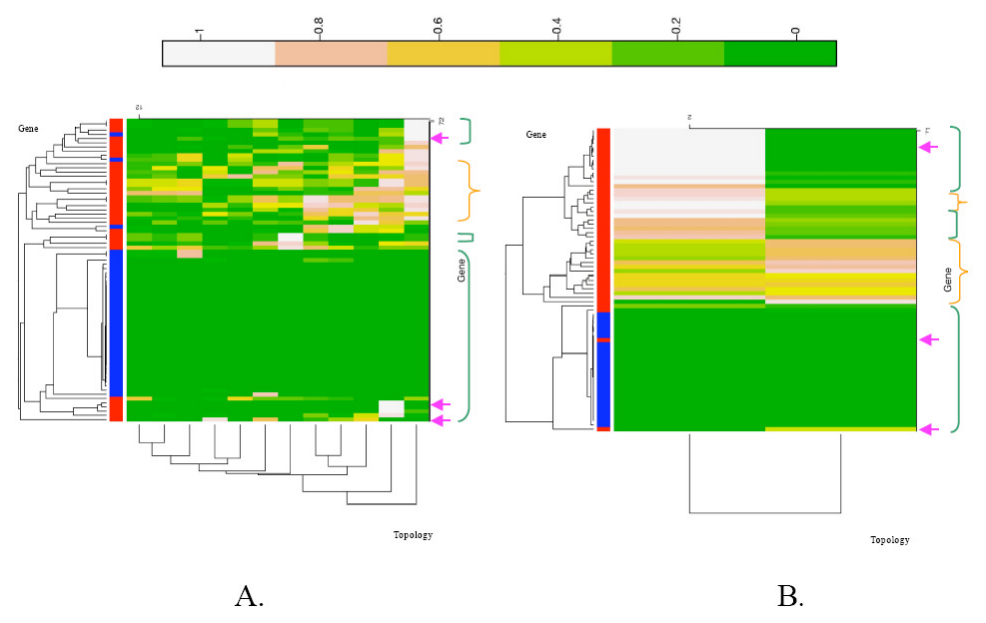
Figure 2A. displays the heat map for the alphaproteobacterial dataset and figure 2B for the bacterial dataset. See the legend of Figure 1 for details.

We feel that our heat maps challenge the use of a tree-like pattern to describe molecular evolution. There was always more than one plausible topology retained (see Additional file 1 for a description of the diversity of these plausible topologies). Archaeal and eukaryotic markers favoured 60 and 92 topologies, respectively (Figures 1A and 1B), and alpha-proteobacterial markers (Figure 2A) favoured 12 different trees. Among those best topologies, none are supported by all the genes. Instead, a given topology is accepted by some markers and rejected by others, leading to multiple multicolour lines in mosaic heat maps. The absence of an entirely light line means that genes fail to agree on a single topology, even though they reject many of them and thus do contain some phylogenetic signal. This seems compatible with the redefined "weak" view of the tree. By contrast, the bacterial markers were apparently more discriminating and retained two plausible trees only (Figure 2B), one of which was supported by most of the actual markers. Furthermore, these two topologies are compatible. Could these results support an organismal tree? In fact, these trees consist of two star-phylogenies, which differ only in their ability to recover the monophyly of proteobacteria, and do not resolve any deep relationships between accepted monophyletic groups otherwise. The absence of basal resolution leaves all possibilities concerning the process of molecular evolution in bacteria open. Such a star-tree can be explained either by multiple ancient LGT events, a radiation, or the lack of ancient phylogenetic signal.

Interestingly, even though many positive controls for LGT are easily identified by the heat map by their propensity to reject the plausible topologies, the discrimination between actual markers and artificial ones is far from absolute. Some artificial genes cannot be distinguished from groups of actual markers (Figures 1B and 2A). In all the heat maps, a significant proportion of weakly discriminating genes is present. We do not know how vertical their phylogenetic history is, because not only do they agree with many different trees, but sometimes they also cluster with artificial markers. Moreover, some actual genes behave as groups of markers with transfers (see Figures 1A and 2B, for instance) and constitute common clusters of markers rejecting most of the plausible topologies. It is tempting to suggest that these genes may have undergone LGT. This would be the case for instance for *rpl37ae*, *rpl15e *in archaea (Figure 1A) or *fmt *in bacteria (Figure 2B).

Finally, independently of the positive controls, actual markers with a strong phylogenetic signal do not necessarily agree. We indicated by pink arrows some instances where conflicting patterns of colour are observed. Every heat map presents several such cases. These disagreements between orthologues and also sometimes the rejection of all plausible trees cannot be taken as evidence that there is a unique true tree for all these genes. We will investivage these cases further in the near future. The message of these heat map analyses is rather that we do not know if orthologues of these datasets share a unique history or not, and there is reason to suspect that they might not.

### Attempted departure from tree-thinking

**Figure 3:**
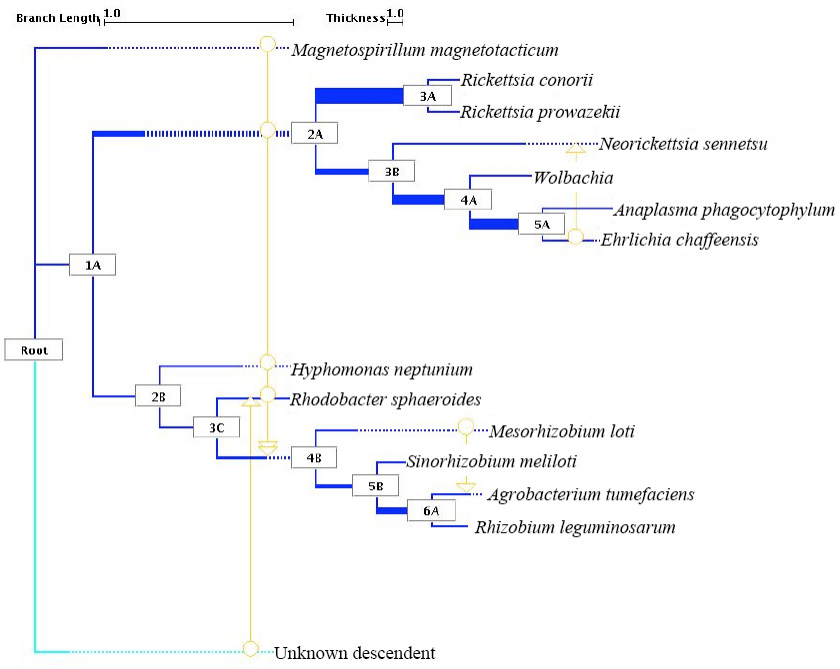
Figure 3 displays the synthesis of 34 alphaproteobacterial genes (*atp1, atp6, atp9, cob, cox2, cox3, nad1, nad2, nad3, nad4, nad4l, nad5, nad6, nad7, nad8, nad11, rpl2, rpl5, rpl6, rpl11, rpl14, rpl16, rpoA, rpoB, rpoC, rps7, rps10, rps12, rps13, rps14, rps19, sdh2, sdh3 *and *tufA*). The proposed vertical-inheritance backbone representing the concatenation tree is shown in dark blue, with the line thickness of an internal branch corresponding to the frequency of its support across the whole dataset. Support was considered significant when clades received > 50% bootstrap support. Putative LGT events are in orange, connecting donors (circles) with recipients (arrowheads); where there are multiple possible donor candidates, these converge onto a double arrowhead. This happens when the clade founded by a past LGT donor may have subsequently had its species membership obfuscated by later exchanges of genetic material, yielding a non-reference assemblage of species labels in a presumed lineage. Where the apparent donor of a gene falls outside of the taxa included in the analysis, one is created as a basal group taxon, indicated in light blue. In order to avoid graphical congestion, branches in the tree may be artificially extended, as dotted segments.

Let's then forget the tree-pattern and briefly consider one instance of what a different but cautious phylogenetic method could teach us. We further explored the dataset of alpha-proteobacteria, for which we had concluded that the presence of LGT could not be rejected nor could the existence of a unique history be proven. We freed ourselves from *a priori *tree-drawing constraints and, with the simple goal of summarizing the safe phylogenetic information of each marker, we obtained a graph that is not a tree. In this synthesis (Figure 3), 25 vertical branches are visible as well as 4 lateral branches. The comparison of the total support for the horizontal and vertical branches indicates that the vertical signal is about 15 times more important than the horizontal signal. A large majority of the genes in this dataset (30/34) does not seem to have been laterally transferred, and many parts of the inferred vertical backbone are relatively robust. However, only node 3A, the clade of Rickettsiales, is supported by all the 34 gene trees. In all the other cases, we would not be right to claim that the vertical backbone corresponds to the common history for all or even most of the markers. For instance, 21 genes do not tell anything about node 2A. Why should we then assume that these 21 markers were subjected to this pattern of vertical inheritance? Certainly, phylogeny alone does not tell us that, and the synthesis shows us clearly that we simply do not know what the history of most genes is, for most of the nodes. Finally, there are still 4 genes (*rps19*, *rps10*, *rpl14 *and *rpoB*) that have likely undergone LGT. LGT between these species occurred only once, thus no generality can be inferred from them, except that 75% of them correspond to local rearrangements of the concatenation tree. More precisely, *rps19 *was transmitted from *Ehrlichia *to *Neorickettsia*, *rpl14 *was transmitted from *Mesorhizobium *to *Agrobacterium *and *rps10 *of *Rhodobacter *apparently comes laterally from a species not studied in this dataset. The origin of the *rpoB *of Rhizobiales is, however, more complex and it cannot be mapped directly onto the reference tree, since multiple donors are possible.

## Conclusion

Heat map analyses are powerful statistical methods to investigate correlations in multiple markers. Certainly, any conclusion deduced from their study depends on the set of topologies, genes and p-values investigated. However, this cannot be seen as a rebuttal in itself to reject this approach. In fact, the same issue arises for any phylogenetic and statistical analyses comparing trees. Importantly, some propositions could be extrapolated from these heat maps applied to the three domains of Life. First, we observed several cases where it was impossible to separate markers with an extremely atypical phylogenetic signal from actual markers. More than a weakness of the method (PCA does the same, data not shown), this might be explained by a relative weakness of the phylogenetic signal contained in many markers. What we report then is simple and sadly not surprising: the genes of eukaryotes and alpha-proteobacteria, for instance, cannot really discriminate between several different topologies. This absence of convergence on a single topology is obviously not evidence for LGT in itself. It is, however, a major issue, since it indicates that with phylogenetic analyses and statistical tools only, we often cannot decide if LGT is present in datasets of orthologues. The broad bacterial and archaeal datasets seem principally free from such extreme recent events. Indeed, in their heat maps, the majority of genes with transfer can be separated from actual markers. There are, however, some instances of LGT in these heatmaps too, since they present clusters of genes with LGT rejecting all the plausible topologies in which some actual markers are also found. Hence, overall, there is no strong phylogenetic evidence that any of these datasets are really comprised of congruent genes.

This could be problematic because phylogeneticists, raised as classical tree-thinkers, often desire a tree or a supertree. We feel that they might be prone to forget/accept the fundamental weaknesses of the markers they use to reconstruct the past. It could matter because if these analyses mix together markers with arguably different histories, the phylogenetic Tree of Life will be simply a phenetic tree, a "Trend of Life," averaging noise, signal, and different histories of markers to fit an *a priori *pattern. In other words, a phylogeneticist who would assume that he had reconstructed the organismal tree from orthologues and produced a genealogy of organisms instead of a central tendency might be a victim of an extreme version of tree-thinking. Yet, no phylogeneticist has to be an extreme tree-thinker anymore, because there is no phylogenetic evidence for that. Consequently, we see the present conclusion as a positive one. In fact, this work should encourage attempts to explore more accurately the phylogeny of organisms. We propose that a safer, more punctual use of the phylogenetic signal of orthologues could be envisioned. On one hand, "*whereof one cannot speak thereof one must be silent*" applies [42], while on the other hand we cautiously and resolutely report all the phylogenetic information that we can. Acknowledging that a strong version of tree-thinking has still to be proven and should not be assumed *a priori*, and that a weak version could be refined, we could reduce the risk of building a hazardous evolutionary history from the largely unknown phylogenetic signal of orthologues. This acknowledgement would also allow us to maximise the number of genes available for phylogenetic analysis, instead of limiting cautious simultaneous analyses to a few congruent markers. It may not produce a tree in the end, but would surely be more grounded.

## Methods

### Alignments and preliminary phylogenetic analyses

One eukaryotic dataset and three prokaryotic datasets (archaea, bacteria, and alpha-proteobacteria) were investigated. The eukaryotic dataset (34 genes:*a-rad51, c-psma, d-rpl12e, e-ef2, rpl10a, rpl10b, rpl11b, rpl13a, rpl15e, rpl19e, rpl28e, rpl37a, rpl1, rpl10, rpl17, rpl18, rpl2, rpl26, rpl27, rpl3, rpl30, rpl9, rps11, rps15, rps16, rps19, rps20, rps23, rps4, rps8, rps15p, rps27e, sap40, srs*, 17 species:*S. cerevisiae, S. pombe, E. cuniculi,,G. theta nucleomorph, P. yezoensis, A. thaliana, O. sativa, C. reinhardtii, D. melanogaster, H. sapiens, C. elegans, D. discoideum, E. histolytica, P falciparum, T. gondii, T. pyriformis *and *P. infestans*) is a subset from Bapteste et *al. *[5] The archaeal dataset (44 informational genes: *rpl10, rpl14p, rpl15e, rpl15p, rpl18e, rpl18p, rpl19e, rpl21e, rpl22p, rpl23p, rpl24e, rpl24p, rpl2p, rpl30p, rpl31e, rpl32e, rpl37ae, rpl3p, rpl40e, rpl44e, rpl4p, rpl5p, rpl6p, rpl7ae, rps10p, rps11p, rps12p, rps13, rps14p, rps15p, rps17e, rps17p, rps19e, rps19p, rps24e, rps2p, rps3p, rps4e, rps4p, rps5p, rps6e, rps7p, rps8e, rps8p*, 18 species:*S. solfataricus, A. pernix, P. aerophilum, M. kandleri, P. abyssi, P. horikoshii, P. furiosus, T. acidophilum, T. volcanium, A. fulgidus, M. maripaludis, M. acetivorans, H. marismortui, M. thermoautotrophicus, S. tokodaii, F. acidarmanus, M. jannashii and Halobacterium sp*.) corresponds to an update of Brochier et *al. *[6], including *Nanoarchaea *and some methanogenic species. The bacterial dataset (45 genes: *efg, fmt, if1, if2, ksga, npt, rba, rf2, rpl1, rpl10, rpl11, rpl14, rpl15, rpl16, rpl17, rpl18, rpl19, rpl2, rpl20, rpl21, rpl23, rpl24, rpl27, rpl29, rpl3, rpl32, rpl34, rpl35, rpl4, rpl5, rpl6, rpl7, rpl9, rps11, rps12, rps13, rps2, rps20, rps3, rps4, rps5, rps6, rps7, rps8, rps9, trmd*, 28 species:*P. gingivalis, C. tepidum, P. marinus, D. radiodurans, B. anthracis, B. subtilis, C. difficile, S. pyogenes, M. leprae, M. tuberculosis, S. coelicolor, T. maritima, A. aeolicus, B. burgdorferi, T. pallidum, C. pneumoniae, C. trachomatis, C. jejuni, H. pylori, C. crescentus, R. capsulatus, R. prowazekii, B. pertussis, N. meningitidis, N. europaea, E. coli, P. aeruginosa *and *V. cholerae*) corresponds to the core of genes identified in Brochier et *al. *[33]. The alpha-proteobacterial dataset (34 genes: see Figure 3 for their name, 13 species) corresponds to orthologous proteins shared by the mitochondria of *Reclinomonas *and all alpha-proteobacteria. All these alignments were inspected, manually refined if required, and are available upon request. For all individual markers, preliminary analyses by NJ using MUST.3.0 [43] and Maximum likelihood (ML) using PROML with the JTT amino acid substitution matrix, a rate heterogeneity model with gamma-distributed rates over four categories with the α parameter estimated using TREE-PUZZLE, global rearrangements and randomized input order of sequences (10 jumbles), were done to exclude potential non-orthologous copies, but no such copies were identified. For each dataset, all the genes were concatenated to calculate a best tree by ML (PROML + JTT model + 9 categories). Lengths of the concatenations were approximately 6300, 7300, 7800, 5900 for archaea, bacteria, alpha-proteobacteria, and eukaryotes, respectively. The best ML tree was calculated for each gene individually by the same methodology.

### Constitution of matrices for statistical analyses

A set of topologies for each dataset was constructed to test the congruence and the phylogenetic signal between markers. They contain alternatives to the best concatenation tree for each dataset. The best ML tree issued from a concatenation of the markers in a dataset was used as a reference and rearranged by moving each species of the tree to any other possible location to simulate recent LGT inside a reference tree. The dataset also included (i) a star topology, (ii) topologies supporting only a single robust monophyletic group, (iii) topologies containing all the accepted monophyletic groups, but also showing one event of LGT. For instance, in case (iii), the clade of *Ferroplasma/Thermosplasma *includes a *Methanosarcina *that should have been located in another clade, under the hypothesis of an organismal reference tree. This approach generated a set of 868/1197/1142 and 443 test topologies for archaea, eukaryotes, bacteria and alpha-proteobacteria, respectively. These input trees are given in Additional file 2. They were used as user trees in TREE-PUZZLE5.1, option -wsl, with a JTT+Γ 8+I model of evolution to estimate the likelihood of each site of a given gene and global tree likelihoods for each tree. These two sets of likelihood values were used as input for CONSEL [44] to perform the Approximately Unbiased (AU) test [45] and associate a p-value to each set of generated trees.

To test if the actual markers behave differently from genes with LGT, datasets of markers presenting different degrees of LGT were generated as follows. We randomly assigned the sequence of one species to another one, as if the latter has just laterally acquired the sequence of the former. After this operation, a gene alignment presents one additional extreme and recent LGT event. We reiterated this up to three times per gene, generating up to three additional LGT events in a single alignment. These alignments are the positive controls for LGT. If the statistics of genes with LGT are identical to those of actual markers, LGT presence cannot be excluded.

### Statistical analyses

Heat map analysis was implemented in R . Heat maps of p-values of the AU test were used to test that genes support similar topologies. A spot with a dark green colour indicates low p-values for a topology tested for a given gene. By contrast, a spot with a light colour indicates high p-values, i.e. good support for this topology by a given gene. These spots of colour can be clustered to highlight the presence of patterns of support/ rejection, by rearranging rows and columns separately for genes and topologies, so that they correspond to a dendrogram from hierarchical clustering. In this way, clusters of genes (topologies) showing similar patterns of support across topologies (genes) are grouped together and easily seen. Hierarchical clustering dendrograms were obtained using the Euclidean distance matrix for the vectors of p-values. The definition of the number of clusters will be discussed in a future paper.

To completely summarize patterns of support for topologies it would be necessary to include all possible topologies. This is impractical for the data sets here (even the set of *a priori *plausible topologies included makes visualization difficult). To utilize the information from tests over a large number of topologies while easing visualization of results, we present heat maps with a restricted set of "plausible" topologies for which the majority of genes had a p-value larger than 0.05. This set of plausible topologies is thus constructed under the hypothesis of interest that genes should share support for a single topology due to their common vertical descent. The set is also larger than required under the hypothesis that genes come from a single topology due to common vertical descent. Under this hypothesis, the p-values for that topology should be uniformly distributed across genes so that 95% of the genes are expected to have p-values larger than 0.05 for this topology. With probability larger than 0.95, at least 90 out of 100 genes should have p-values larger than 0.05 for the correct topology. Thus the set of plausible topologies could be restricted to the set with p-values greater than 0.05 for 80 to 90% of the genes. Since, for the datasets considered here, restriction to topologies with a majority of p-values larger than 0.05 eased visualization sufficiently, we did not make further restrictions. Note that the full set of topologies is being utilized, since with a larger set of initial topologies, a larger set of plausible topologies will be found. In principle, the initial set of topologies should be large enough that all topologies satisfying the criteria of plausibility are included.

### Synthesis reconstruction

The synthesis of alpha-proteobacteria [32] was inferred from the analyses of the 34 ML trees for 13 species. ML trees were calculated as described above. Their bootstrap support values represent a consensus (obtained using CONSENSE) of 100 Fitch-Margoliash distance trees (obtained using PUZZLEBOOT and FITCH) from pseudo-replicates (obtained using SEQBOOT) of the original alignment. The settings of PUZZLEBOOT were the same as those used for PROML, except that global rearrangements and randomized input order of sequences are not available in this program. PROML, CONSENSE, FITCH and SEQBOOT are from the PHYLIP package version 3.6a . PUZZLEBLOOT can be obtained from the TREE-PUZZLE website . The clades supported with more than 50% bootstrap support in these 34 gene trees were compared to the concatenation tree of alpha-proteobacteria using two programs: Horizstory and Lumbermill [46]. These programs can be downloaded from . Briefly, Horizstory allows inference of the most parsimonious scenarios involving LGT and vertical descent to explain the common features and the discrepancies between the concatenation tree and each of the 34 gene trees. Lumbermill draws the synthesis by mapping the outcomes of these scenarios onto the reference tree. A strict consensus option was applied, meaning that only the relationships supported or inferred in 100% of the evolutionary scenarios resulting from the comparison between the reference and a given tree were considered in this drawing.

## Authors' contributions

EB did the phylogenetic analyses, provided the ideas and wrote the paper, ES implemented the heat map analyses and provided ideas, JL created the alphaproteobacterial dataset, DM implemented Lumbermill, RLC implemented Horizstory, WFD provided ideas.

## Supplementary Material

Additional File 1 The diversity of plausible topologies Click here for file

Additional File 2 Input trees for the AU test Click here for file
