# Supplementary material for: Do orthologous gene phylogenies really support tree-thinking?
Source: BMC Evol Biol. 2005 May 24;5:33. doi: 10.1186/1471-2148-5-33 (PMC1156881; doi:10.1186/1471-2148-5-33)
Supplement: Additional File 2 — Input trees for the AU test [file 1471-2148-5-33-S2.ppt]

## Slide 1
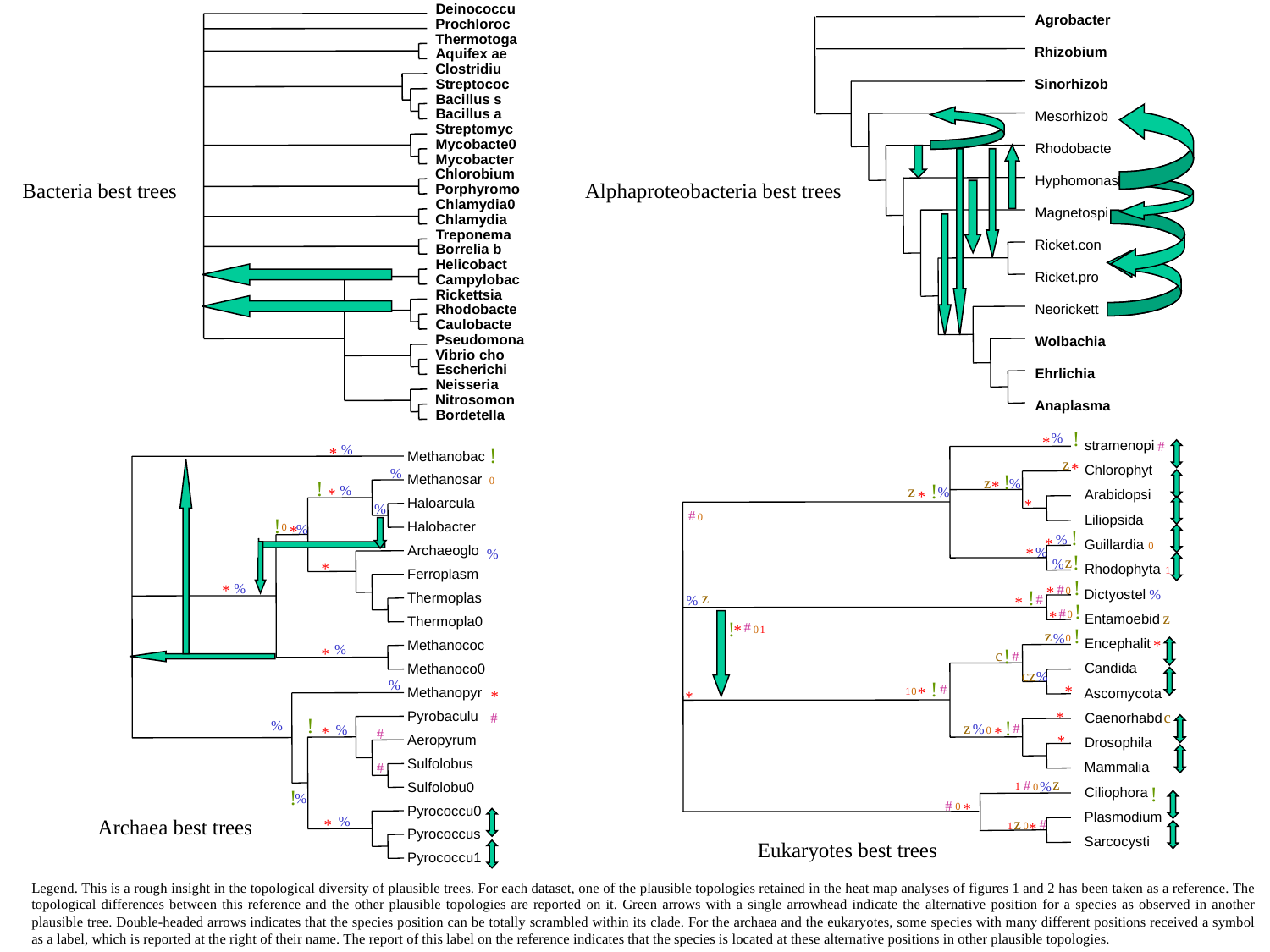

Deinococcu
Agrobacter
Prochloroc
Thermotoga
Rhizobium
Aquifex ae
Clostridiu
Streptococ
Sinorhizob
Bacillus s
Bacillus a
Mesorhizob
Streptomyc
Mycobacte0
Rhodobacte
Mycobacter
Chlorobium
Bacteria best trees
Alphaproteobacteria best trees
Hyphomonas
Porphyromo
Chlamydia0
Magnetospi
Chlamydia
Treponema
Ricket.con
Borrelia b
Helicobact
Ricket.pro
Campylobac
Rickettsia
Neorickett
Rhodobacte
Caulobacte
Pseudomona
Wolbachia
Vibrio cho
Escherichi
Ehrlichia
Neisseria
Nitrosomon
Anaplasma
Bordetella
!
%
*
#
%
!
*
stramenopi
Methanobac
Methanosar
Haloarcula
Halobacter
Archaeoglo
Ferroplasm
Thermoplas
Thermopla0
Methanococ
Methanoco0
Methanopyr
Pyrobaculu
Aeropyrum
Sulfolobus
Sulfolobu0
Pyrococcu0
Pyrococcus
Pyrococcu1
z
*
%
Chlorophyt
!
z
0
%
!
*
!
%
z
%
*
*
Arabidopsi
*
%
#
0
!
Liliopsida
%
0
*
!
%
*
0
Guillardia
%
*
%
!
z
%
*
1
Rhodophyta
!
%
#
*
*
0
!
%
z
#
%
Dictyostel
*
!
#
*
0
z
!
Entamoebid
#
*
!
0
1
z
%
0
*
%
Encephalit
!
*
c
#
c
z
Candida
%
%
!
#
*
*
1
0
*
*
Ascomycota
*
c
#
!
!
Caenorhabd
%
z
#
%
%
*
*
0
#
*
Drosophila
#
Mammalia
z
#
%
1
!
0
!
%
Ciliophora
#
*
0
%
Archaea best trees
z
Plasmodium
*
#
*
1
0
Eukaryotes best trees
Sarcocysti
Legend. This is a rough insight in the topological diversity of plausible trees. For each dataset, one of the plausible topologies retained in the heat map analyses of figures 1 and 2 has been taken as a reference. The topological differences between this reference and the other plausible topologies are reported on it. Green arrows with a single arrowhead indicate the alternative position for a species as observed in another plausible tree. Double-headed arrows indicates that the species position can be totally scrambled within its clade. For the archaea and the eukaryotes, some species with many different positions received a symbol as a label, which is reported at the right of their name. The report of this label on the reference indicates that the species is located at these alternative positions in other plausible topologies.
